# Localization of (+)-Catechin in *Picea abies* Phloem: Responses to Wounding and Fungal Inoculation

**DOI:** 10.3390/molecules25122952

**Published:** 2020-06-26

**Authors:** Tuula Jyske, Katsushi Kuroda, Susanna Keriö, Andrey Pranovich, Riikka Linnakoski, Noriko Hayashi, Dan Aoki, Kazuhiko Fukushima

**Affiliations:** 1 Natural Resources Institute Finland (Luke), Production Systems Unit, Tietotie 2, 02150 Espoo, Finland; 2 Forestry and Forest Products Research Institute, 1 Matsunosato, Tsukuba 305-8687, Japan; kurodak@affrc.go.jp (K.K.); hayashin@ffpri.affrc.go.jp (N.H.); 3 Department of Forest Sciences, University of Helsinki, P.O.B 27, 00014 Helsinki, Finland; susanna.kerio@ct.gov; 4 Connecticut Agricultural Experiment Station, Department of Forestry and Horticulture, 123 Huntington Street, P.O. B 1106, New Haven, CT 06504-1106, USA; 5 Åbo Akademi University, Johan Gadolin Process Chemistry Center, Porthaninkatu 3, 20500 Turku, Finland; apranovi@abo.fi; 6 Natural Resources Institute Finland (Luke), Natural Resources Unit, Latokartanonkaari 9, 00790 Helsinki, Finland; riikka.linnakoski@luke.fi; 7 Nagoya University, Department of Forest and Environmental Resources Sciences, Furo-cho, Chikusa-ku, Nagoya 464-8601, Japan; daoki@agr.nagoya-u.ac.jp (D.A.); kazu@agr.nagoya-u.ac.jp (K.F.)

**Keywords:** axial phloem parenchyma, defense response, fungal inoculation, morphological changes, phenolics, tannins, tissue-specific chemical mapping

## Abstract

To understand the positional and temporal defense mechanisms of coniferous tree bark at the tissue and cellular levels, the phloem topochemistry and structural properties were examined after artificially induced bark defense reactions. Wounding and fungal inoculation with *Endoconidiophora polonica* of spruce bark were carried out, and phloem tissues were frequently collected to follow the temporal and spatial progress of chemical and structural responses. The changes in (+)-catechin, (−)-epicatechin, stilbene glucoside, and resin acid distribution, and accumulation patterns within the phloem, were mapped using time-of-flight secondary ion mass spectrometry (cryo-ToF-SIMS), alongside detailed structural (LM, TEM, SEM) and quantitative chemical microanalyses of the tissues. Our results show that axial phloem parenchyma cells of Norway spruce contain (+)-catechins, the amount of which locally increases in response to fungal inoculation. The preformed, constitutive distribution and accumulation patterns of (+)-catechins closely follow those of stilbene glucosides. Phloem phenolics are not translocated but form a layered defense barrier with oleoresin compounds in response to pathogen attack. Our results suggest that axial phloem parenchyma cells are the primary location for (+)-catechin storage and synthesis in Norway spruce phloem. Chemical mapping of bark defensive metabolites by cryo-ToF-SIMS, in addition to structural and chemical microanalyses of the defense reactions, can provide novel information on the local amplitudes and localizations of chemical and structural defense mechanisms and pathogen–host interactions of trees.

## 1. Introduction

Conifers have foundational roles in many terrestrial ecosystems and in the global forest industry. The boreal forests in the Northern Hemisphere contain approximately 30% of the global carbon stock [1], making them a significant carbon sink. Climate change is predicted to increase the frequency of abiotic and biotic disturbances that affect conifer health [2,3], which will likely have an impact on the dynamics of carbon sequestration in the boreal forests. Norway spruce (*Picea abies* (L.) Karst.) is an important boreal forest tree species, which is predicted to suffer from climate change due to increasing droughts and more frequent outbreaks of the spruce bark beetle (*Ips typographus* L) [2].

Due to their longevity, conifers have evolved to fend off pests and pathogens with a plethora of defense mechanisms. Conifers integrate multiple bark defense mechanisms against biotic invaders and abiotic stressors, including preformed (constitutive) and inducible defense compounds and mechanical (structural) barriers [4,5,6]. Especially under suboptimal conditions, maintaining these defenses is metabolically costly [7,8] and may reduce the trees’ tolerance to other disturbances. For instance, pathogen attack can reduce conifer drought tolerance. On the other hand, drought may alter the resistance of trees to insects and pathogens [9,10]. Against this background, understanding the dynamics of chemical tree defense reactions in Norway spruce is paramount.

Among the microbes that damage trees, bark and ambrosia beetle-vectored fungal pathogens pose a high risk to tree health, especially if introduced to new areas where they are not native. These pathogenic fungi affect trees by clogging vessels and tracheids in stems and roots, which is visible as dark staining of the sapwood and often result in rapid tree death. The causal agents of Dutch elm disease (*Ophiostoma* spp.) [11], laurel wilt (*Raffaelea lauricola* T.C. Harr., Fraedrich and Aghayeva) [12], black stain root disease (*Leptographium spp*.) [13], pine blue-stain (*Grosmannia clavigera* (Rob.-Jeffr. and R.W. Davidson) Zipfel, Z.W. de Beer, and M.J. Wingf.) [14], and *Fusarium* dieback (*Fusarium euwallaceae* S. Freeman, Z. Mendel, T. Aoki, and O’Donnell) [15], are all insect-vectored fungi that cause severe damage to their host trees. In the Eurasian boreal region, *Endoconidiophora polonica* (Siemaszko) Z.W. de Beer, T.A. Duong and M.J. Wingf. is a highly virulent, endemic blue-stain fungus of Norway spruce [6,16]. The fungus is vectored by *I. typographus*, which causes more damage to Norway spruce during low rainfall years [17,18]. The predicted increases in summertime droughts and temperatures in the boreal forests indicate a higher risk for bark beetle infestations [2], which could also increase *E. polonica* infections.

The amplitude and localization of the chemical defense responses of Norway spruce against ophiostomatoid fungi are not completely understood. Norway spruce bark contains high concentrations of several polyphenolic defense compounds, including stilbene glucosides, flavonoids, and tannins. Norway spruce phloem contains up to 10% (DW) of stilbene glucosides [19,20,21,22,23]. These phenolic secondary metabolites show a wide range of biological activities, such as the antifungal and antibacterial properties vital to the bark resistance to pathogens. In the bark of Norway spruce, (+)-catechin and (−)-epicatechin are found as subunits (i.e., flavan-3-ol units) of procyanidins, which are the most abundant type of condensed tannins (CTs), also known as proanthocyanidins (PAs) [24,25]. CTs are the only tannins that occur in coniferous tree species [26].

In several plant species, catechins have been found to provide protection against, for example, oxidative stress, herbivore attacks, and bacterial invasions. In conifers, catechin production is induced in response to inoculation with *E. polonica* [27,28,29], ophiostomatoid fungi [28,30], and other fungal pathogens [31]. Within the last decade, new light has been shed on catechin biosynthesis and functions as defensive compounds in Norway spruce [31,32,33,34]. For instance, Norway spruce genotypes that produce higher amounts of (+)-catechin are more resistant to the root rot pathogen *Heterobasidion parviporum* [34]. Bioassay analyses have revealed that (+)-catechins clearly inhibit the growth of *E. polonica* when present in concentrations similar to those produced in bark as a response to fungal inoculation, providing evidence in support of the defensive role of (+)-catechins in spruce bark [33]. Interestingly, *E. polonica* can catabolize Norway spruce phenolic compounds, including (+)-catechin *in vitro* and cause a decline of the phenolic compounds astringin and isorhapontin [21,22,23] in infected Norway spruce tissue [35]. This reflects the ability of *E. polonica* fungus to cope with the chemical defenses of conifers [35], which may be important for the survival of bark beetles in the host trees [36] and highlights the importance of understanding the dynamics and localization of chemical host defenses.

In our recent study, cryo-time-of-flight secondary ion mass spectrometry (cryo-ToF-SIMS) was used to reveal the localization and accumulation patterns of stilbene glucosides within frozen-hydrated phloem (i.e., *in planta* [37]). Cryo-ToF-SIMS can visualize small organic/inorganic compounds in freeze-fixed biological samples at a microscopic resolution [38,39]. As a result, axial phloem parenchyma cells were found to accumulate stilbenes, the amount of which varied with changes in the axial parenchyma cell type and cell volume from the inner to outer phloem. The results confirmed earlier findings suggesting axial parenchyma cells as the main site of accumulation for both preformed and inducible polyphenolics [32]. Axial parenchyma is formed as tangential sheets during annual phloem formation [4,40,41] and enlarged after fungal attack [6]. 

Several studies have monitored spruce bark chemical defense reactions using bulk tissue samples, while limited work has examined the time-dependent chemical and structural reactions at the tissue and cellular levels. To elucidate the tissue and cellular-level bark defenses (both preformed and inducible defenses), it is essential to gather positional information on defense metabolites in a living state, as pretreatments of specimens, such as drying, may change the distributions and concentrations of such compounds [32,42,43].

The aim of the present study was to investigate the tissue and cellular-level localizations of phenolics in Norway spruce phloem before and after wounding and fungal infection caused by *E. polonica* inoculation. We hypothesized that (**1**) (+)-catechins are localized in axial phloem parenchyma cells similarly to stilbene glucosides and that (**2**) their constitutive accumulation pattern across the phloem is similar to that of stilbenes. Moreover, we hypothesized that (**3**) the tissue and cellular-level distribution and accumulation patterns of (+)-catechins and stilbene glucosides change in response to wounding and fungal inoculation. At the same time, we expected to see (**4**) temporal and spatial changes in the tissue-level distribution and accumulation patterns of oleoresin (i.e., resin acids such as abietic acid). Complementary novel techniques, including chemical mapping by cryo-ToF-SIMS-SEM, quantitative chemical microanalysis by GC and GC-MS, and optical (LM) and electron microscopy (TEM), were used. This is a parallel study to that of Jyske et al. [37], which targeted the localization of stilbene glucosides within Norway spruce phloem.

## 2. Materials and Methods

### 2.1. Plant Material and Experimental Design

This study was comprised of two mature Norway spruce trees growing in a forest site and twelve grafted saplings of Norway spruce in a greenhouse experiment. The mature trees were used to quantitate and localize constitutively produced (+)-catechins across the tissues and cells of phloem. The saplings were used to study the effects of wounding and fungal inoculation on the accumulation and distribution of catechins, stilbene glucosides, and oleoresin compounds.

**Figure 1:**
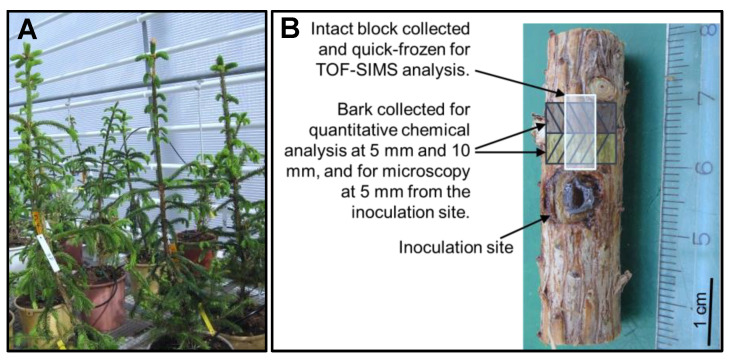
Saplings in the greenhouse experiment (**A**) used to study the wounding and fungal inoculation-induced responses in terms of the amounts and localization of (+)-catechin, stilbene glucosides, and resin acids within the phloem. Stem section containing the inoculation site (**B**) and a schematic presentation of tissue positions from which the specimens for chemical and anatomical analyses of the phloem were collected.

The mature trees were of different ages, and both grew in southern Finland (Loppi; 60°44′N, 24°30′E, 120 m a.s.l.; [37]). The younger tree was 18 years old (12 cm in diameter at 1.3 m height on the stem and 9.4 m in height), and the older tree was 37 years old (29 cm in diameter and 22.1 m in height). Both trees represented different clones of good growth and quality performance and originated from southern Finland. The trees were grown on fertile old agricultural soil. The trees were harvested in October of 2011. Sample discs were sawn at breast height, and bark blocks (ca. 3 × 2 × 2 cm in the longitudinal, radial, and tangential directions, including the whole bark, phloem, and cambium, and a few xylem rings) were immediately cut from the southern sides of the discs (Figure 1). A subsample of each block was rapidly frozen in dry ice (−78 °C), kept in dry ice during transportation to the laboratory, and stored at −80 °C [37].

Six-year-old Norway spruce saplings (mean average height and diameter: 90 ± 11 cm and 13 ± 1 mm, respectively) were used to study the wounding and fungal inoculation-induced responses in terms of the amounts and localization of (+)-catechins and other defense compounds within the phloem. The saplings represented clones of good growth and quality performance, and the grafts originated from Poland. The saplings were grafted and planted at an outdoor plot at the tree breeding center of the Natural Resources Institute Finland in south Finland (Loppi; 60°44′N, 24°30′E, 120 m a.s.l.) in 2009. In early May of 2015, the saplings were transferred into containers and placed into a greenhouse to adjust for two weeks prior to the onset of the inoculation treatment (see Figure 1A). Inoculations of the saplings were performed on the 19th of May, at two weeks before the onset of their shoot growth. The saplings were randomly split into three treatments: unwounded controls (C; altogether four saplings), wounded controls (W; two saplings), and fungal-inoculated saplings (F; six saplings). Along the lower half of the main stem, four bark plugs (7 mm Ø, including cambium) were removed with a cork borer. For the wounded controls, plugs of sterile malt agar were placed into the wounds and enfolded with parafilm (W4, two saplings). The fungus-treated stems were divided into two subgroups: two saplings were treated with (1) four plugs of *Endoconidiophora polonica* isolate (CBS 142282) with previously demonstrated pathogenicity [16], and four saplings were treated with (2) two plugs of malt agar and two plugs of *E. polonica* culture (W2F2) to study the within-plant responses to wounding and fungal inoculation. The parallel inoculation sites within the stem were located on the opposite sites of the stem. The vertical distance between the sites was ca. 10 cm.

Two control saplings (C) were harvested on the day of inoculation treatment (day 0); two wounded-inoculated (W2F2) saplings were harvested at the days 7 and 17 after inoculation (i.e., DAI 7 and 17); and two control saplings (C), two wounded saplings (W4), and two inoculated samplings (F4) were sampled at 23 DAI. Small strips of bark were collected next to the wound (at the distances of 1–5 mm and 5–10 mm from the inoculation site) for quantitative chemical analysis, quick-frozen in liquid nitrogen LN_2_, and stored at −80 °C (see Figure 1). For the *in planta* chemical mapping of compounds within the phloem and bark, an intact disc of ca. 5 cm long (in the vertical direction along the stem axis) was collected (including the inoculation/wounded site on the lower end of the specimen) from the parallel inoculation/wounded site on the stem, quick-frozen in liquid nitrogen LN_2_, and stored at −80 °C (Figure 1).

### 2.2. ToF-SIMS Analysis of (+)-Catechins, Abietic Acid, and Stilbenes

First, we localized the constitutive (+)-catechins within the phloem of mature trees using the same specimens as those used to study the localization of stilbene glucosides [37]. Second, we analyzed the localization and accumulation of (+)-catechin, stilbenes, and resin acids within the phloem of the saplings after wounding and fungal inoculation by using methodology shown in [37,43]. Briefly, frozen-hydrated samples were cut smaller (ca. 5 × 5 × 7 mm) and kept frozen with liquid N_2_. Then, the specimen was freeze-attached to the sample holder of the cryo-ToF-SIMS/SEM system, and its surface was cut evenly using a sliding microtome (REM-710, Yamato Koki, Japan). A special cryo-stage was used (ca. −130 °C) to prevent ice sublimation during the analysis.

ToF-SIMS measurements were carried out using a TRIFT III spectrometer (ULVAC-PHI, Chigasaki, Japan). The pressure in the ToF-SIMS specimen chamber was ca. 1 × 10^−8^ Pa. Positive spectra were obtained by using 22 keV Au_1_^+^ ions at a current of 5–7 nA. The measured surface areas were 200 × 200 µm^2^ and 500 × 500 µm^2^, with a sample surface primary ion dosage of <2.3 × 10^9^ and secondary ion counts of >2 × 10^6^. Sequential images of the phloem surface were obtained by joining overlapping ToF-SIMS images. Each image (raw data) contained a full mass spectrum with a resolution of 256 × 256 pixels. The mass spectrum data for the estimated candidate peaks of (+)-catechin, abietic acid, and stilbenes within the phloem were analyzed as spectral data and images.

**Figure 2:**
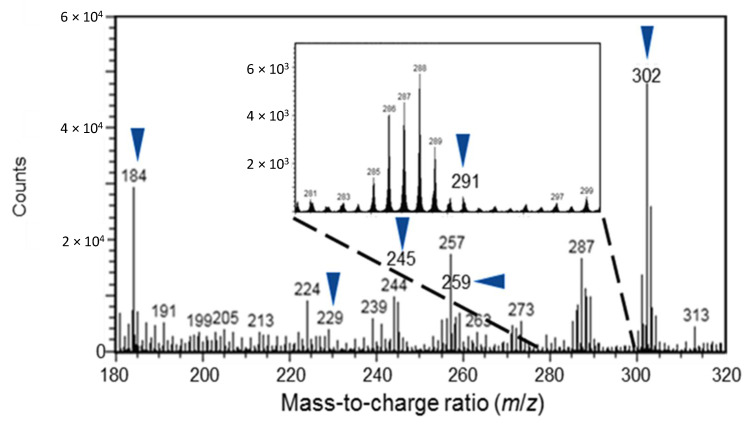
A positive ToF-SIMS spectrum of the frozen-hydrated, non-treated, control Norway spruce phloem of a sapling. Arrows indicate the peaks generated from (+)-catechin and abietic acid (*m*/*z* of 291).

The compounds within the phloem were identified by the direct comparison of their peaks at the corresponding mass-to-charge ratio (*m*/*z*) with the peaks of authentic compounds, as analyzed by ToF-SIMS (Figure 2, Figure S1; [37]). We used authentic piceid, resveratrol, piceatannol, isorhapontigenin (Sigma-Aldrich, Saint-Louis, MO, USA), astringin, isorhapontin (Polyphenols Laboratories AS, Sandnes, Norway), (+)-catechin, and abietic acid (both Sigma-Aldrich, Saint-Louis, MO, USA). The standard spectra of the authentic compounds under dried conditions were measured by TOF-SIMS (both spectral and image-mode data; see spectra in Figure S1). To obtain the standard spectra of the compounds in frozen-hydrated conditions, we prepared their aqueous solutions with 0.1 M potassium chloride (KCl; 0.4 mg mL^−1^ aq. KCl). The frozen solutions were then measured with cryo-ToF-SIMS. The positive ion was selected for the analysis, and repeatable results were obtained.

For the spatial analysis of the compound distribution and accumulation, the corresponding peak intensities at the estimated *m*/*z* were detected, correlated to the total secondary ion counts (i.e., relative peak intensities) in each image, and presented as per mille of the total ion counts [37]. The region of interest (ROI) function of the WinCadence program (ULVAC-PHI Inc., Chigasaki, Japan, version 5.1.2.8) was applied to manually isolate specific tissue and cell regions in each image to produce and compare mass spectral data between these ROIs. The obtained ToF-SIMS images were connected using WinCadence 5.1.2.8 (ULVAC-PHI Inc., Chigasaki, Japan) and MATLAB R2014a (The MathWorks, Inc., Natick, MA, USA) with PLS Toolbox 7.5.2 (Eigenvector Research, Inc., Manson, WA, USA), without any ion count normalization. The color scale for the obtained united image was changed using ImageJ software (The National Institutes of Health, Bethesda, MD, USA, http://imagej.nih.gov/ij/). The line profiles of selected ions were estimated using ImageJ software. After cryo-ToF-SIMS analysis, the phloem samples were transferred to cryo-SEM using a cryo-vacuum shuttle and attached to the cryo-stage of the SEM to verify their frozen status during the ToF-SIMS analysis. Secondary electron images were obtained at an accelerating voltage of 3 kV [43]. To observe the structural details through freeze etching, the temperature of the stage was raised to around −85 °C with constant scanning and then cooled to −130 °C after reaching the required level of etching (Figure S5).

### 2.3. Cryo-Sectioning and GC Analysis of (+)-Catechins and Stilbenes in Older Trees

The quantitative amounts of (+)-catechin and stilbenes [37] were analyzed in the bark of mature Norway spruce trees. The frozen blocks were cut into smaller subblocks (ca. 10 × 10 × 10 mm) on dry ice, and serial tangential cryo-sections (250 or 450 µm each) were cut from the outermost bark towards the cambial zone using a cryo-microtome (−25 °C). The cryo-sections were placed in Eppendorf vials and immediately freeze-dried. The freeze-dried phloem tissue was weighed and extracted with 3 mL of acetone–water (9:1 *v/v*) in a test tube at room temperature in the dark (total 24 h) three times. The contents of the test tube were periodically agitated. The clear extract was transferred to another test tube with a Pasteur pipette, and the solvent from the cumulative extract was evaporated in a water bath at 40 °C with N_2_ flow and carefully dried in a vacuum desiccator at 40 °C for 1 h in the dark. The dried extractive content was determined, and 10 mL of acetone was added to each test tube to obtain stock solutions of the extractives. An aliquot of the stock solution was placed in a separate test tube to obtain approximately 1 mg of extractives; xylitol and betulinol were added as standards (100 µL of 0.1 mg/mL xylitol in methanol and 2 mL of 0.02 mg/mL of betulinol in methyl tert-butyl ether (MTBE), respectively); and the solvents were evaporated under N_2_ flow. Silylation was performed with a mixture of BSTFA-TMCS-pyridine (4:1:1 *v/v/v*) at 70 °C for 40 min, and the sample was left overnight in the dark to complete derivatization. The silylated sample was then analyzed by GC-FID using a PerkinElmer AutoSystem XL Gas-Chromatograph (PerkinElmer, Waltham, MA, USA) equipped with HP-1 (25 m × 0.2 mm; 0.11 μm film thickness) and HP-5 (25 m × 0.2 mm; 0.11 μm film thickness) capillary columns, with the temperature programming providing simultaneous analyses of mono-/disaccharides and stilbene glucosides during one GC run [37]. The sample injection volume was 1 μL, and the split ratio was 1:30. Monosaccharides and disaccharides were quantified against xylitol using correction factors determined by the separate analysis of authentic mono-/disaccharides. The extractives, including (+)-catechins, were quantified against betulinol without using a correction factor (i.e., CF 1.0). The quantitative results were presented for each cryo-section as a function of the cumulative distance from the cambium.

### 2.4. Quantitative GC-MS Analysis of the Phloem Defense Compounds of Saplings

The tissue-level changes in phloem defense compounds after wounding and fungal inoculation of Norway spruce saplings were analyzed using GC-MS (Figure 1). Bark strips were collected from the wounding and/or inoculation point on the stem and cut into matchstick-sized pieces, freeze-dried for 72 h, and milled with a ball mill (Retsch MM 400, Retsch GmbH, Haan, Germany) while being kept frozen. Approximately 20 mg of powder was extracted with an acetone–water solution (95:5, *v/v*, including 0.2 mg mL^−1^ of heptadecanoic acid as an internal standard) in an ultrasonic water bath (USC300TH, VWR, Radnor, PA, USA) for 30 min. After sonication, the tubes were centrifuged at 3000 rpm for 5 min, and 1 mL of supernatant of each sample was pipetted into an autosampler vial. The samples were evaporated with N_2_, silylated with 0.5 mL of *N*-trimethylsilyl imidazole (TMSi in pyridine, Sigma-Aldrich, Merck KGaA, Darmstadt, Germany) and kept in a heater at 60 °C for 30 min or at room temperature overnight before analysis. The silylated extracts were analyzed by GC–MS (Agilent Hewlett-Packard 6890 and Hewlett-Packard 5973 MSD, EIMS 70 eV; Agilent, Santa Clara, CA, USA) equipped with a Zebron ZB SemiVolatiles capillary column, as described in [23]. Quantitative analysis of the identified compounds ((+)-catechin, epicatechin, stilbene glucosides astringin, isorhapontin, piceid, and resin acids) was performed using an internal standard (heptadecanoic acid, C:17) and authentic compounds as external standards.

### 2.5. Phloem Microscopic Analysis

From the sapling stems, small specimens in the transverse and radial directions (5 × 1 × 5 mm in radial × tangential × longitudinal) were immediately cut off and placed in a test tube containing a fixative solution (freshly made mixture of 5% glutaraldehyde−8% paraformaldehyde in 0.1 M phosphate buffer; pH 7.4). The fixation time of the specimens was ca. 4 h at room temperature (RT). The specimens were then washed in 0.1 M phosphate buffer and postfixed with 2% osmium tetroxide for 1 h at RT. They were then washed again in phosphate buffer, dehydrated with a graded series of ethanol, incubated with a transitional solvent (acetone), and gradually embedded in low-viscosity resin (TAAB, Reading, UK). Semithin sections (500 nm) were first prepared and stained with toluidine blue and then pre-examined with an optical microscope to select the area of interest for TEM analysis. From the selected specimen area, ultrathin transverse and radial sections (60 nm; covering the phloem, the cambial zone, and part of the developing xylem) were cut and stained with uranyl acetate and lead citrate and examined with a Jeol JEM-2000EX TEM at an accelerating voltage of 80 kV. Semithin sections were analyzed with a light microscope (BX 50, Olympus, Tokyo, Japan), and images were taken with a digital camera (PixeLINK, Ottawa, ON, Canada).

### 2.6. Statistical Analysis

The statistical significance of the differences in catechins, stilbene glucosides, and resin acids between treatments was analyzed by mixed-model analysis (IBM SPSS Statistics 22; IBM Corporation, Armonk, NY, USA) after first testing the assumption of a normal distribution of data. The fixed terms in the model were treatment (i.e., unwounded control, wounding, and fungal inoculation), time (i.e., days after treatment onset), and location (i.e., distance from the treatment site on the stem), while sapling was introduced as a random term. Multiple pairwise comparisons between treatments, days and distances were adjusted by Bonferroni corrections. The differences in the relative ion intensities (i.e., the relative ion intensities of the estimated compounds to the total ion counts) between different cell types (i.e., the axial parenchyma and intermediate spaces including sieve cells and ray parenchyma) were analyzed by an analysis of variance (ANOVA, IBM SPSS Statistics 22; IBM Corporation, Armonk, NY, USA) after first testing the assumption of homogeneity of the variances.

## 3. Results

### 3.1. Tof-SIMS Spectrum of Norway Spruce Phloem

A typical positive spectrum of Norway spruce phloem obtained from the middle of a sapling specimen is shown in Figure 2. An example ToF-SIMS spectrum of authentic standard compounds ((+)-catechin and abietic acid) is shown in Figure S1. The spectral peaks that were discovered from both the authentic compounds and the phloem specimens were selected as estimated peaks representing (+)-catechins and abietic acid (a major resin acid compound of spruce bark, representing oleoresin compounds), with the *m*/*z* values of 291 and 302, respectively. The *m*/*z* 301 and 302 ions showed nearly the same distribution and were probably both derived from abietic acid. The fragment ions of stilbene glucosides at *m*/*z* 229, 245, and 259 were selected to represent piceid, astringin, and isorhapontin, as previously described [37]. To identify living cells, we detected the head group fragment ion of phosphatidylcholine (PC; [C5H15NPO4]^+^), the major phospholipid component of cell membranes [44,45,46,47,48] at *m*/z 184) and 302, respectively.

### 3.2. Localization and Accumulation of Constitutive (+)-Catechin within Phloem

The constitutive amounts and localization of (+)-catechins within the phloem of mature trees were analyzed by observing the same specimens as those used to study the localization of stilbene glucosides [37]. To analyze the distribution and accumulation patterns of (+)-catechin in relation to the corresponding patterns of stilbene glucosides, mapping data of *m*/*z* 291, 229, 245, and 259 were obtained from the cambium to the outer bark. Additionally, mapping data of *m*/*z* 184 was acquired, representing the location of living cells.

**Figure 3:**
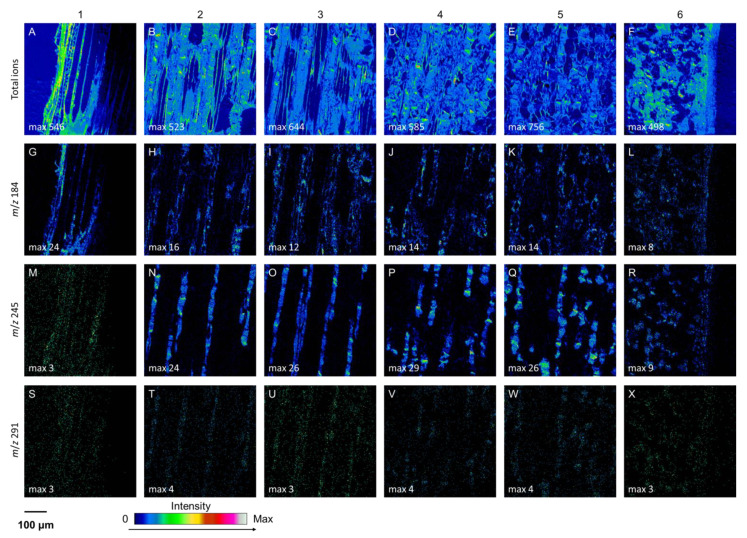
Positive ToF-SIMS images at the radial-longitudinal surface of the phloem in an older, non-treated, healthy Norway spruce tree from the cambium and non-collapsed phloem on the left (**1**) to the inner (**2**), middle (**3**–**4**), and outer (**5**) regions of the collapsed phloem, and the outermost collapsed phloem and periderm (**6**) on the right. Total secondary ions (**A**–**F**), *m*/*z* at 184 (**G**–**L**), 245 (**M**–**R**), and 291 (**S**–**X**) represent tissue structures, membranes of living cells, trans-astringin, and (+)-catechin, respectively.

**Figure 4:**
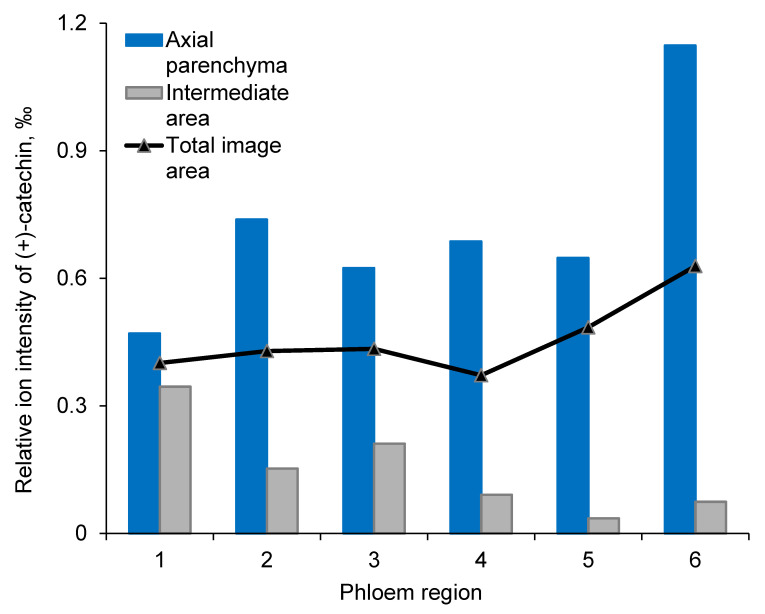
Relative ion intensity of (+)-catechin at *m*/*z* 291 as detected by ToF-SIMS in the regions of interest (ROI) in a non-treated, healthy tree. Axial phloem parenchyma (blue bars) and intermediate areas (gray bars) composed of sieve cells and ray parenchyma at the radial-longitudinal phloem surface of an older Norway spruce tree from the cambium and non-collapsed phloem on the left (**1**) to the inner (**2**), middle (**3**–**4**), and outer (**5**) regions of the collapsed phloem, with the outermost collapsed phloem and periderm (**6**) on the right. The black line represents the relative ion intensity of (+)-catechin in the total image area in each phloem region. The phloem regions are the same as those shown in Figure 3. The relative ion intensities were computed per mL of the total ion counts in each image or ROI. The ion intensities of (+)-catechin were statistically higher in the axial parenchyma than in the intermediate spaces (*p* < 0.001).

At the tissue and cell levels, ToF-SIMS analysis revealed similar localization and accumulation patterns of (+)-catechins to those of stilbene glucosides (Figure 3, Figures S2 and S3). The compounds were localized and compartmentalized inside the living axial phloem parenchyma cells (Figure 3 and Figure 4, and Figure S2). In the regions of interest (ROIs) of the axial parenchyma, the relative ion intensity of (+)-catechin was significantly (*p* < 0.001) higher than in the ROIs representing intermediate areas (i.e., areas composed of sieve cells and ray parenchyma; Figure 4). In the youngest, innermost phloem, the localization of (+)-catechin was not clearly separated between axial parenchyma and intermediate areas due to immature tissues (see region 1 in Figure 4).

**Figure 5:**
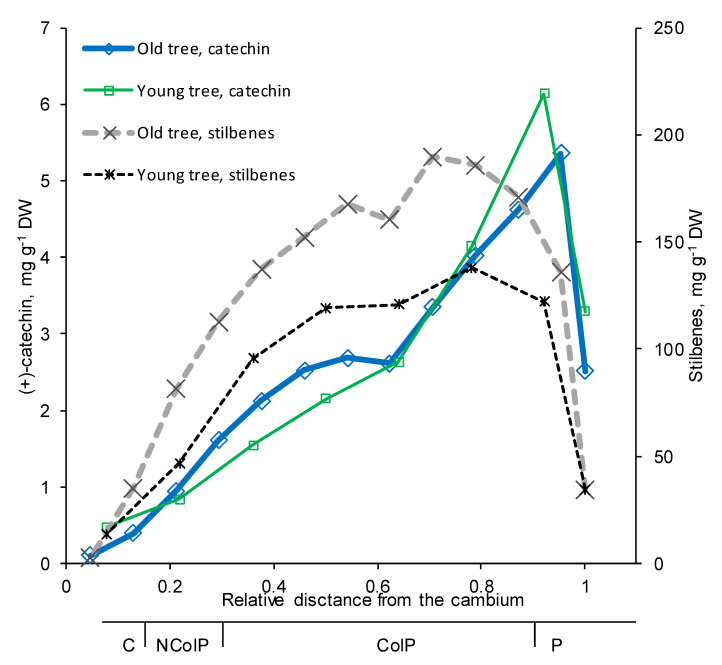
Constitutive radial changes in (+)-catechin and stilbene glucoside amounts (sum of astringin and isorhapontin) across the mature Norway spruce phloem of an older and a younger non-treated tree, as analyzed by GC-FID. Each data point represents a tangential section (250–450 μm thick) representative of different phloem zones: C, cambium; NColP, noncollapsed phloem; ColP, collapsed phloem; P, periderm.

The quantitative GC and GC-MS analysis of tangential cryo-sections of phloem showed that the (+)-catechin content of mature Norway spruce phloem increased from the inner to the outer phloem, reaching the highest amount next to the cork cambium (in phelloderm; Figure 5). In dead periderm, the (+)-catechin content was clearly decreased (Figure 5). The radial patterns of (+)-catechin amounts within phloem were similar between younger (17-year-old) and older (38-year-old) trees. In contrast, stilbene glucosides astringin and isorhapontin showed higher levels of accumulation with increasing tree age (Figure 5).

### 3.3. Anatomical Changes in Phloem after Wounding and Fungal Inoculation

**Figure 6:**
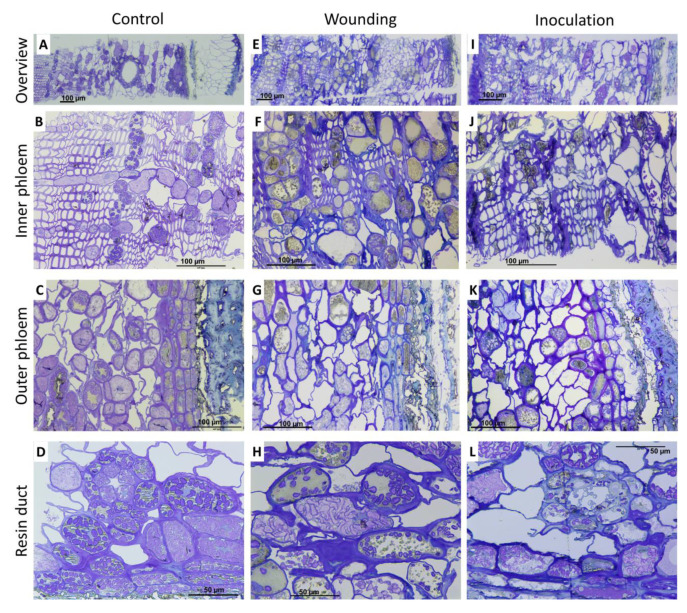
Bright field microscopy images of transverse surface of phloem in saplings of Norway spruce 23 days after inoculation: control saplings (**A**–**D**), wounded saplings (**E**–**H**), and fungus-inoculated saplings (**I**–**L**). The uppermost panels (**A,E,I**) show overview of phloem; panels B, F, and J show the tissues in innermost phloem (tangential rows of axial phloem parenchyma, sieve cells); the panels C, G, and K show the outermost phloem; and panels D, H, and L show the details of cellular properties. Scale bars on each figure.

The anatomical changes and the localization and accumulation of phenolic compounds in Norway spruce phloem in response to wounding and fungal inoculation were analyzed. The patterns of (+)-catechin, stilbene glucosides, and resin acids were analyzed to follow changes in the chemical and structural defenses in tissues and individual cells. At the time of treatment onset, the secondary growth of both phloem and xylem was active (data not shown). In response to fungal inoculation, a rapid cessation of secondary growth and the formation of collapsed, desiccated necrotic phloem tissues were observed at 7 DAI. The phloem tissues of inoculated saplings showed accumulations of vacuolar and cell wall-bound dark and electron-dense (phenolic) substances at 17 and 23 DAI (Figure 6 and Figure S4). Wounding induced the differentiation of cambial cells into ray parenchyma-type cells, callus formation, and wound healing (data not shown).

### 3.4. Phloem Chemical Defenses after Wounding and Fungal Inoculation

**Figure 7:**
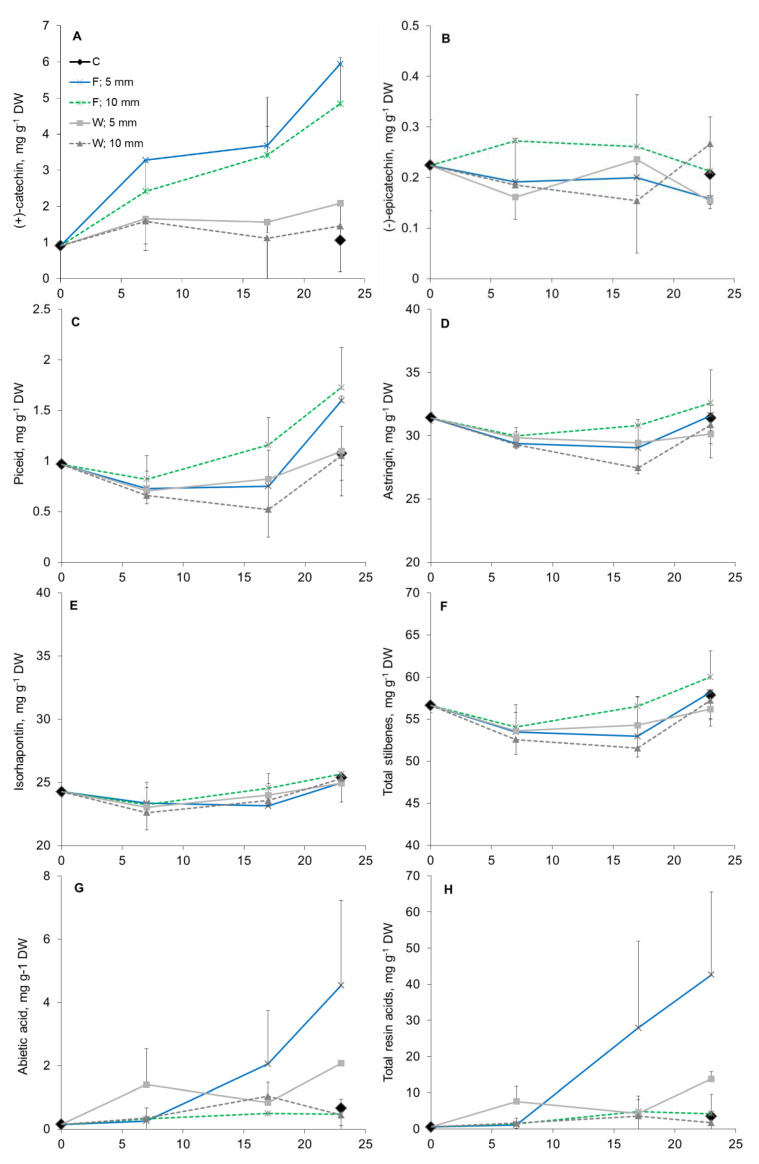
Amounts of (+)-catechin (**A**), (−)-epicatechin (**B**), stilbene glucosides piceid (**C**), astringin (**D**), isorhapontin (**E**), total stilbenes (**F**), abietic acid (**G**), and total resin acids (H) in the phloem and bark of Norway spruce saplings during the 23-day period after treatment onset, as analyzed by GC-MS. The treatments were: control (“C”), wounding (“W”), and inoculation with fungi (“F”). Samples were at 5 and 10 mm from the treated location (wound/inoculation).

A clear quantitative chemical response to fungal inoculation was observed at 7 DAI. The (+)-catechin content significantly increased in the inoculated trees compared to those in wounded and control specimens and continued to rise during the course of the 23-day experiment (Figure 7, Table 1). The (+)-catechin content was higher closer to the inoculation site (up to 5 mm) compared to those at more distant locations (from 5 to 10 mm from the inoculation site; *p* < 0.08) (Table 1). No treatment effect on the (−)-epicatechin content was detected.

Fungal inoculation caused increased the content of stilbene glucosides piceid and astringin compared to control and wounding treatments (Figure 7, Table 1). The total stilbene glucoside amount first decreased (7 DAI) and then increased from 17 to 23 DAI. The piceid amount at 23 DAI was higher (*p* < 0.01) in fungus-infected than in control and wounded specimens. Fungal inoculation increased the flow of resinous compounds by 1.8-fold and 5.7-fold compared to those in the wounding and control treatments, respectively, especially in the vicinity of the treated tissue and towards the end of the experiment (Table 1 and Table 2, Figure 7).

### 3.5. Mapping of Defense Compounds Within Phloem after Wounding and Fungal Inoculation

**Figure 8:**
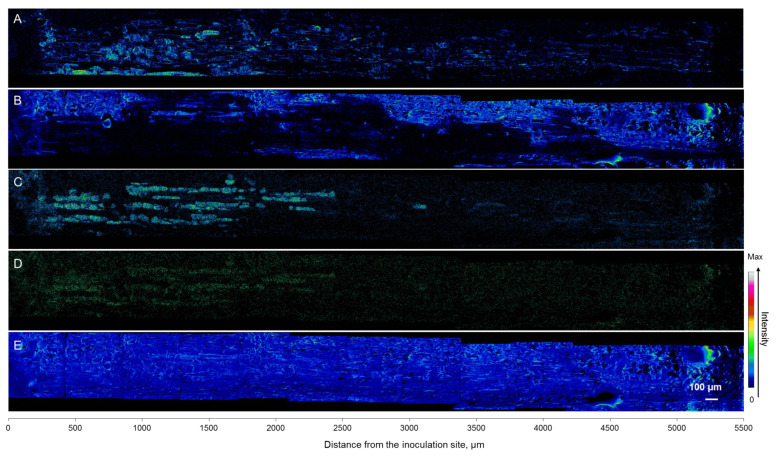
Positive ToF-SIMS images of Norway spruce phloem from the inoculation site (on the left) upwards along the sapling stem (to the right). The outer bark is at the upper side of each image. Images of *m*/*z* at 184 (**A**), 302 (B), 245 (**C**), and 291 (**D**) represent living cells, abietic acid, stilbene glucoside astringin, and (+)-catechin, respectively. Total ion images (**E**) represent the structures of the sample surface. The colors of the pixels correspond to the ion intensities of the compounds on the specimen’s surface. Specifically, abietic acid, (+)-catechin, and stilbenes were absent from dark areas, showed mid-range concentrations in blue and green areas, and had the highest concentrations in yellow and red areas.

**Figure 9:**
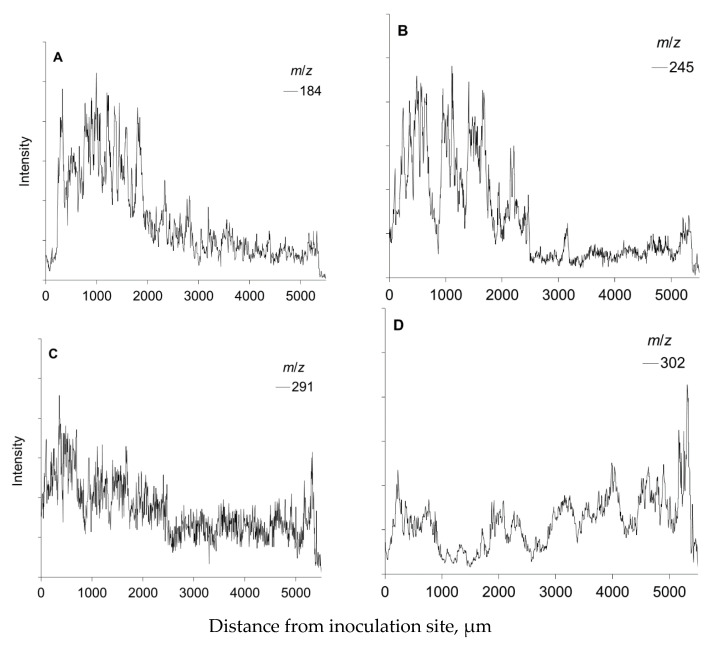
Ion intensities of *m*/*z* at 184 (**A**), 245 (**B**), 291 (**C**), and 302 (**D**) representing living cell membranes, stilbene glucoside astringin, (+)-catechin, and abietic acid, respectively, obtained from ToF-SIMS spectra of the radial-longitudinal surface of a frozen-hydrated, fungus-inoculated Norway spruce sapling. The specimen is the same as that shown in Figure 8. The ion intensity values of three consecutive pixels were averaged, and the results are shown as a function of the distance (µm) from the inoculation site.

At the cellular level, (+)-catechins and stilbene glucosides were localized in the axial phloem parenchyma. Their accumulation significantly increased towards the inoculation site, as clearly observed by ToF-SIMS mapping and changes in the ion intensities of the compounds (Figure 8 and Figure 9). Interestingly, the signal from living cells at *m*/*z* 184 also increased towards the inoculation site (Figure 8 and Figure 9), following the distribution and accumulation patterns of stilbene glucosides and (+)-catechins. No direct *in planta* visualization was obtained to show that phenolic substances in the axial phloem parenchyma cells colocated with *E. polonica* hyphae.

**Figure 10:**
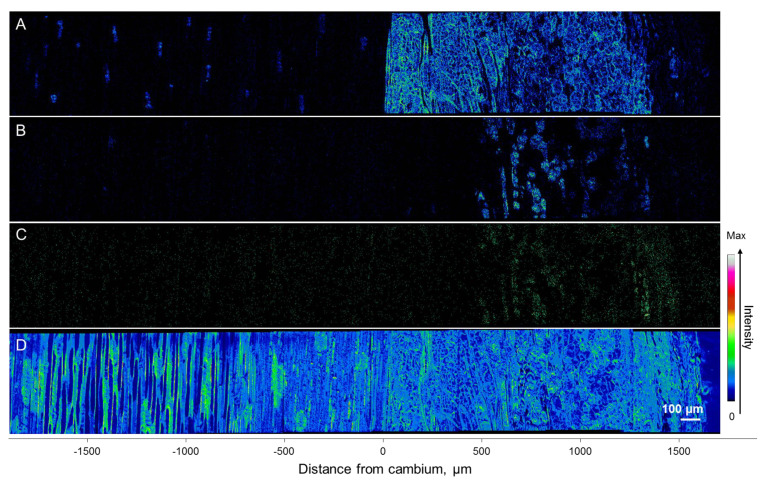
Positive ToF-SIMS images at the tangential-radial surface of wounded Norway spruce phloem from the xylem (on left) to the outer bark (on right) at different distance from the cambium (µm) The specimen location is 4 mm upwards from the wounding site on the sapling stem. Secondary ions at *m*/*z* 184 (A), 245 (**B**), and 291 (**C**) represent the membranes of living cells, stilbene glucoside astringin, and (+)-catechin, respectively. The total secondary ions (**D**) represent the tissue structures. The color of the pixels corresponds to the ion intensities of the compounds on the specimen surface. Specifically, (+)-catechin and stilbene glucosides were absent from dark areas, showed mid-range concentrations in blue areas, and had the highest concentrations in green and yellow areas. K areas showed mid-range concentrations in blue areas, had the highest concentrations in green and yellow areas.

**Figure 11:**
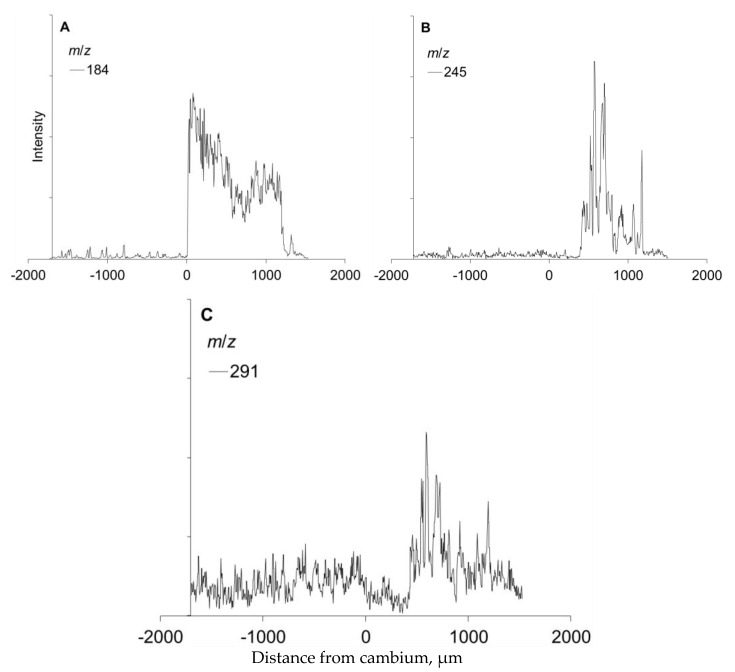
Ion intensities at *m*/*z* 184, 245, and 291 representing the membranes of living cells (**A**), stilbene glucoside astringin (**B**), and (+)-catechin, as detected by ToF-SIMS from the tangential-radial surface of frozen-hydrated Norway spruce phloem at different distance (µm) from the xylem to the cambium (the cambium location is 0 on the x-axis) and from the cambium to the outer bark of a wounded sapling. The ion counts are for the images shown in Figure 10**.**

In wounded specimens, (+)-catechins and stilbene glucosides were not induced or present in the newly formed callus tissue (Figure 10 and Figure 11; at the distance of 0–500 µm from the cambium towards the outer bark). The patterns of polyphenolic compounds from the cambium to the outer layers of the cortex and epidermal tissue closely followed those detected in mature spruce phloem (Figure 10 and Figure 11 vs. Figure 5).

Oleoresin compounds (i.e., represented by abietic acid) were detected as being spread at the outer layer of the cortex, with the localization mainly not overlapping with the axial parenchyma and their phenolic substances (Figure 8). During the experiment, we did not detect the formation of traumatic resin canals. The quantitatively analyzed resin acid content was highly variable between saplings and specimens (Figure 7). The variation of abietic acid was also observed in ToF-SIMS analysis (Figure 8 and Figure 9).

## 4. Discussion

### 4.1. (+)-Catechins are Localized in Living Axial Parenchyma Cells in Norway Spruce Phloem

To advance forestry practices and develop more efficient ways to prevent different pathogen-derived damages, it is important to understand the mutual interplay between fungi and the local amplitudes and localizations of chemical defense responses of host trees. In Norway spruce, the axial phloem parenchyma cells, i.e., polyphenolic parenchyma cells, have key roles as the sites of storage, accumulation, and synthesis of phenolic compounds [32,37,40,49], which have important roles both in constitutive and inducible defenses against pests and pathogens [4,6,40]. In this study, we applied the novel topochemical mapping tool cryo-ToF-SIMS-SEM to study the (+)-catechin localization patterns within the phloem. We found strong evidence confirming our hypothesis that (+)-catechin is localized in axial phloem parenchyma cells with a similar distribution pattern to that of stilbene glucosides [37]. The constitutive accumulation pattern of (+)-catechin across the phloem was very similar to that of stilbenes, showing an increase in amount from the cambium outwards, followed by a decrease in dead periderm tissues (Figure 5). The constitutive levels of (+)-catechin monomers and polymers in the bark of mature Norway spruce trees vary from 0.1 to 9 mg g^−1^ per dry weight (DW) [21], whereas in young Norway spruce trees the levels are highly variable (0.5–25 mg g^−1^ DW) [33]. In our study, the (+)-catechin levels were low to moderate for non-wounded healthy mature trees (0 to ca. 6 mg g^−1^ DW), and low for the young non-wounded healthy saplings (ca. 1 mg g^−1^ DW). These low constitutive levels highlight the role of (+)-catechins in induced defense.

We observed (+)-catechin and stilbenes being specifically localized in the axial phloem parenchyma, suggesting that these cells are the primary location for their storage and synthesis in Norway spruce. The localization of (+)-catechin and stilbenes did not change during the experiment, and they were detected inside the axial phloem parenchyma in wounded and fungus-infected samples, similarly to the control samples. Based on this, it appears that the (+)-catechins are not translocated to the ray parenchyma in the xylem and phloem nor to the tissues formed after wounding and fungal inoculation. The axial phloem parenchyma seems to be symplasmically isolated from the conducting sieve elements and their accompanying Strasburger cells [50]. However, they are apparently connected to ray parenchyma, based on the pits found in their interconnected cell walls [50]. Based on the localization of phenylalanine ammonia lyase, a key enzyme of the phenylpropanoid pathway, synthesis of polyphenolic compounds has been suggested to occur both in the axial parenchyma cells and ray parenchyma [40,49]. However, the complete absence of the ion intensities corresponding to stilbenes astringin and (+)-catechin in phloem rays and the limited connectivity between axial parenchyma and ray parenchyma [50] indicates that these compounds are synthesized only in the axial phloem parenchyma. This highlights the importance of these cells as a source of defensive compounds [37,40].

### 4.2. Induced Tissue and Cell-Level Responses to Wounding and Fungal Infection

After fungal inoculation with *E. polonica*, stilbene biosynthesis has been shown itself to be upregulated, while the amounts of stilbene glucosides decreased and stilbene aglycons increased [32,51,52]. In this study, compared to the unwounded control trees (saplings), the (+)-catechin amount doubled in the wounded trees, whereas *E. polonica* inoculation resulted in a 6-fold increase in (+)-catechin content. In line with our results, the (+)-catechin content (including monomers, dimers and polymers) has been shown to significantly increase in response to *E. polonica* inoculation in Norway spruce bark both in 8-year-old saplings [33] and in mature trees [27,53,54]. Additionally, higher (+)-catechin synthesis has also been associated with higher resistance to the root-rotting fungus *Heterobasidion annosum* s.l. [31,34]. Additionally, *in vitro* studies indicate that stilbene glucosides inhibit the fungal growth of *E. polonica* [52,55,56]. This supports the idea that (+)-catechin has an important role in conifer defense against fungal pathogens.

In our study, the total stilbene glucoside content first slightly decreased until 14 DAI, after which the piceid and astringin contents slightly increased in wounded and infected phloem. The decrease in the stilbene glucoside content has been shown to be due to the fungal transformation of phenolics; i.e., fungus can utilize stilbenes and catechins as energy sources [35,36,52]. Spruce bark beetles have been shown to prefer growth medium amended with bark defense phenolics after being modified by *E. polonica* overgrowth medium with unmodified phenolics [36]. These findings suggest that bark beetle-associated fungi may have important roles in facilitating the survival and persistence of the beetles in the host trees.

We hypothesized that the tissue and cellular-level distributions and accumulation patterns of (+)-catechin and stilbene glucosides change in response to wounding and fungal inoculation. This assumption was partly correct, as both stilbene and (+)-catechin accumulation within the axial phloem parenchyma greatly increased towards the wounded and infected tissue zones. In contrast, the stilbene glucoside content declined in the dead necrotic lesion areas adjacent to the point of fungal inoculation. The low (+)-catechin concentrations in dead tissues further suggest that (+)-catechin is locally synthesized and stored in the axial parenchyma cells. Our results and earlier observations indicate that the living polyphenolic parenchyma cells act as a localized layer of bark defense [32,40].

We also detected an increased signal from living cells at *m*/*z* 184 towards the inoculation site, following the distribution and accumulation patterns of stilbene glucosides and (+)-catechins. This was possibly due to the defense reactions of the living phloem cells but may have also been partly due to the hyphal growth of *E. polonica* (data not shown). *E. polonica* has been observed to grow in the lumens of axial phloem parenchyma and ray parenchyma already at 7 and 14 DAI [49]. However, we did not detect hyphae inside the parenchyma cells. Thus, we did not obtain direct *in planta* imaging results to show how *E. polonica* fungus can catabolize phenolic compounds [35]. However, *E. polonica* has been reported to grow into and through large phenolic deposits in Norway spruce phloem [40,49]. It is possible that the Norway spruce genotypes used in the current study were only moderately susceptible to the used fungal strain, and therefore the fungal colonization was not extensive. The young saplings used in our study had a few layers of uniform tangential strands of axial phloem parenchyma which increased in size in response to fungal inoculation, which is typical for Norway spruce genotypes resistant to *E. polonica* [40].

Fungal infection also led to ample oleoresin flow [57]. We found that abietic acid spread from the resin ducts upon infection and wounding. Abietic acid was localized in morphologically distinct cell layers as compared to axial parenchyma containing polyphenolics. Terpenoid resins are synthesized and stored in specialized epithelial cells of the resin ducts. Together with constitutively produced stilbenes, resin provides the first line of defense after the breakage of the bark surface, allowing time for the formation of inducible chemical and structural defenses against invaders [6]. Hydrophilic phenolics and lipophilic resin compounds are differently located within the phloem and bark, and optimally block fungal hyphae growth and the dehydration of wounded structures [58].

Recently, we showed different visual appearances of the vacuolar phenolic contents of axial phloem parenchyma due to aging of the phloem cells from the cambium towards the outer bark [37]. This was probably due to the condensation, polymerization, and possible degradation of diverse secondary metabolites [59,60] being compartmentalized inside the vacuoles. Catechins of spruce bark polymerize into CTs, having vacuolar accretions that constitute the typical form of tannin storage [61]. The quantification of CTs is complicated by the diversity of the structures, the lack of commercial standards, and the difficulties of resolving high-molecular weight oligomers and polymers of CTs [62,63]. CTs bind proteins, and should the bound protein be an enzyme, CTs block the catalytic sites of the enzyme and inhibit its activity [64]. Tannins can also inhibit the formation of fungal spores [65] and interfere with the synthesis of fungal melanin [66], which is an important virulence factor of several pathogenic fungi [67].

For the resistance capacity of a tree upon fungal attack, earlier studies have stressed the importance of the diversity of constitutive phenolics in the phloem and the tree capacity to induce phenol and flavonoid synthesis, and the formation of insoluble compounds upon attack [29,53,54]. The preformed concentration of defense phenolics presumably has a key role in the ability of the tree to resist pathogen mass attacks [37]. Due to the effects of global changes, spruce trees have become more vulnerable to assaults by bark beetles and their vectored pathogenic fungi [2]. Pathogen-attacked trees have a higher consumption of assimilated carbon due to increases in the repairing costs of disrupted tissues, and increases in induced, carbon-expensive defense metabolites. Fungal infection can cause a decreased xylem sap surface tension, which makes the tree vulnerable to embolism formation and hydraulic failure [Paljakka et al., unpublished data]. Simultaneously, the tree maintains the local carbon-intensive defenses and the repair of disrupted tissues. Thus, pathogens may advance drought-induced tree mortality by directly depleting nonstructural carbon (NSC), by accelerating NSC consumption by the host, or by increasing repairing costs [68,69,70] recently examined how Norway spruce trees allocate newly fixed carbon resources for growth and respiration, carbon storage, and future survival by means of producing secondary defense metabolites. According to their results, spruce has a conservative allocation strategy, in that newly assimilated carbon can be directed to produce secondary metabolites to ensure future survival, whereas NSC for growth and respiration are downregulated during water depletion or carbon limitation. These observations are important for estimating spruce growth and resilience in the changing climate.

## 5. Conclusions

This study showed for the first time *in planta* chemical mapping of (+)-catechin storage and synthesis in Norway spruce phloem. The results demonstrate that the axial phloem parenchyma cells are the primary location for (+)-catechin storage and synthesis in Norway spruce phloem; in response to fungal inoculation, the (+)-catechin amount locally increases. Chemical mapping of metabolites provides novel information on defense mechanisms and pathogen-host interactions of trees. To advance forestry practices and develop more efficient ways for preventing pathogen-derived damages in changing climate in boreal region, it is important to understand the mutual interplay between fungi and host tree tissues, and the local amplitude and localization of chemical defense responses of host trees. Complementary novel micro-techniques, including cryo-time-of-flight secondary-ion mass-spectrometry (cryo-TOF-SIMS-SEM), were shown to be powerful tools to study positional and temporal changes in metabolite distributions within living tissues.

## Figures and Tables

**Table 1:** Mixed model results from testing the differences between treatments (i.e., unwounded control, wounding, and fungal inoculation with *Endoconidiophora polonica*), time after treatment onset (day), and location on the stem (i.e., distance from the treated site: 5 mm and 10 mm) in terms of the quantitative amounts of (+)-catechin, (−)-epicatechin, stilbene glucosides, and resin acids in the phloem and bark of Norway spruce saplings.

Dependent Variable	Fixed Effect	*F*-Value	*p*-Value
(+)-catechin	Treatment (T)	62.91	**0.000**
	Day (D)	3.05	0.110
	Location (L)	5.95	**0.026**
	T*D	4.58	**0.030**
	T*L	0.06	0.814
	D*L	0.21	0.814
	T*D*L	1.02	0.381
(−)-epicatechin	T	1.90	0.193
	D	0.13	0.936
	L	1.76	0.206
	T*D	1.45	0.282
	T*L	0.48	0.499
	D*L	1.52	0.252
	T*D*L	1.12	0.353
Piceid	T	12.27	**0.002**
	D	12.24	**0.000**
	L	0.19	0.669
	T*D	2.29	0.126
	T*L	3.47	0.077
	D*L	0.01	0.990
	T*D*L	1.10	0.350
Astringin	T	4.66	**0.043**
	D	3.18	0.146
	L	0.45	0.511
	T*D	0.97	0.404
	T*L	2.98	0.103
	D*L	0.37	0.697
	T*D*L	1.04	0.378
Isorhapontin	T	0.30	0.589
	D	5.33	0.007
	L	0.31	0.584
	T*D	0.07	0.930
	T*L	0.83	0.373
	D*L	0.32	0.733
	T*D*L	0.35	0.709
Total stilbenes	T	3.15	0.091
	D	6.27	**0.003**
	L	0.36	0.553
	T*D	0.28	0.761
	T*L	2.45	0.133
	D*L	0.25	0.781
	T*D*L	0.96	0.401
Abietic acid	T	1.11	0.307
	D	1.62	0.287
	L	24.97	**0.000**
	T*D	2.21	0.153
	T*L	1.79	0.200
	D*L	6.26	0.010
	T*D*L	3.10	0.073
Total resin acids	T	5.62	**0.029**
	D	1.52	0.309
	L	19.10	**0.000**
	T*D	3.04	0.081
	T*L	3.40	0.083
	D*L	3.81	0.044
	T*D*L	2.11	0.153

“*”: Interaction between fixed factors of the statistical model.

**Table 2:** Resin acid content (arithmetic mean ± standard deviation) in the bark of young Norway spruce saplings in different treatments (i.e., unwounded control, wounding, and fungal inoculation) during the period of 23 days after the treatment onset.

Resin Acid, mg g^−1^ DW	Days after Onset	Treatment				
		Control	Wounding		Fungal Inoculation	
			Location			
			5 mm	10 mm	5 mm	10 mm
Pimaric	0	-				
	7	n.d.	0.14 ± 0.05	-	-	-
	17	n.d.	0.25	-	0.51 ± 0.04	0.05
	23	0.03 ± 0.02	0.20 ± 0.06	-	0.92 ± 0.64	0.11
Isopimaric	0	0.14 ± 0.06				
	7	n.d.	1.57 ± 1.35	0.41 ± 0.53	0.22 ± 0.06	0.33 ± 0.36
	17	n.d.	0.74 ± 0.59	0.68 ± 0.01	2.01 ± 1.67	0.42 ± 0.09
	23	0.83 ± 0.76	2.43 ± 0.39	0.40 ± 0.05	4.21 ± 3.35	0.58 ± 0.72
Palustric	0	0.03				
	7	n.d.	0.45 ± 0.19	0.27	0.08	-
	17	n.d.	0.56 ± 0.76	0.43 ± 0.07	7.35 ± 6.33	0.90 ± 1.03
	23	1.19 ± 0.91	2.02 ± 0.91	0.07	10.58 ± 5.57	1.48
Levopimaric	0	-				
	7	n.d.	0.83 ± 0.09	0.13	0.18	0.24
	17	n.d.	1.02 ± 1.68	0.26 ± 0.03	17.6 ± 3.82	1.97 ± 2.64
	23	0.37 ± 0.48	2.13 ± 1.67	-	9.96 ± 0.95	0.41
Dehydroabietic	0	0.13 ± 0.06				
	7	n.d.	2.54 ± 0.72	0.30 ± 0.31	0.35 ± 0.02	0.20 ± 0.20
	17	n.d.	0.76±0.58	0.36±0.07	2.18 ± 1.79	0.61 ± 0.41
	23	0.74 ± 0.63	3.48 ± 0.58	0.60 ± 0.39	9.01 ± 7.93	1.99 ± 2.66
Neoabietic	0	0.11 ± 0.07				
	7	n.d.	0.64 ± 0.65	0.66	0.13 ± 0.02	0.41 ± 0.44
	17	n.d.	0.50 ± 0.39	0.78 ± 0.06	2.24 ± 1.87	0.41 ± 0.01
	23	0.58 ± 0.55	1.42 ± 0.30	0.23 ± 0.03	3.38 ± 1.79	0.12 ± 0.10
Abietic acid	0	0.15 ± 0.07				
	7	n.d.	1.41 ± 1.13	0.36 ± 0.43	0.26 ± 0.09	0.33 ± 0.35
	17	n.d.	0.84 ± 0.64	1.04 ± 0.14	2.06 ± 1.69	0.50 ± 0.02
	23	0.67 ± 0.55	2.08 ± 0.06	0.45 ± 0.00	4.54 ± 2.70	0.47 ± 0.48
Total resinacids	0	0.50 ± 0.17				
	7	n.d.	7.57 ± 4.20	1.60 ± 2.03	1.09 ± 0.02	1.38 ± 1.52
	17	n.d.	4.26 ± 3.74	3.56 ± 0.10	27.90 ± 24.01	4.83 ± 4.22
	23	3.48 ± 3.60	13.76 ± 2.03	1.71 ± 0.51	42.59 ± 22.92	4.16 ± 5.37

“-“: Amount was below the detection level; n.d., no sample for detection.

## Supplementary Materials

The Supplementary Materials are available online.
