# Supplementary material for: Localization of (+)-Catechin in Picea abies Phloem: Responses to Wounding and Fungal Inoculation
Source: Molecules. 2020 Jun 26;25(12):2952. doi: 10.3390/molecules25122952 (PMC7356009; doi:10.3390/molecules25122952)
Supplement: Supplementary file 1 [file molecules-25-02952-s001.pdf]

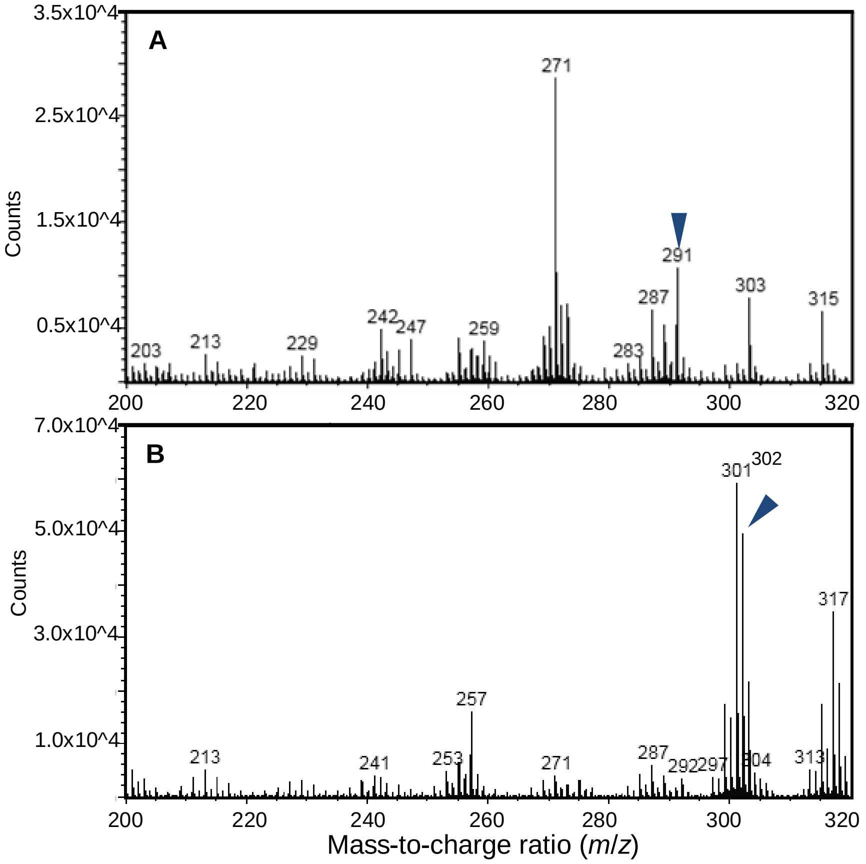

Figure S1. Positive ToF-SIMS spectra of authentic standard compounds in dry condition: (+)-catechin (A); and abietic acid (B). The peaks of interest are indicated with arrowheads.

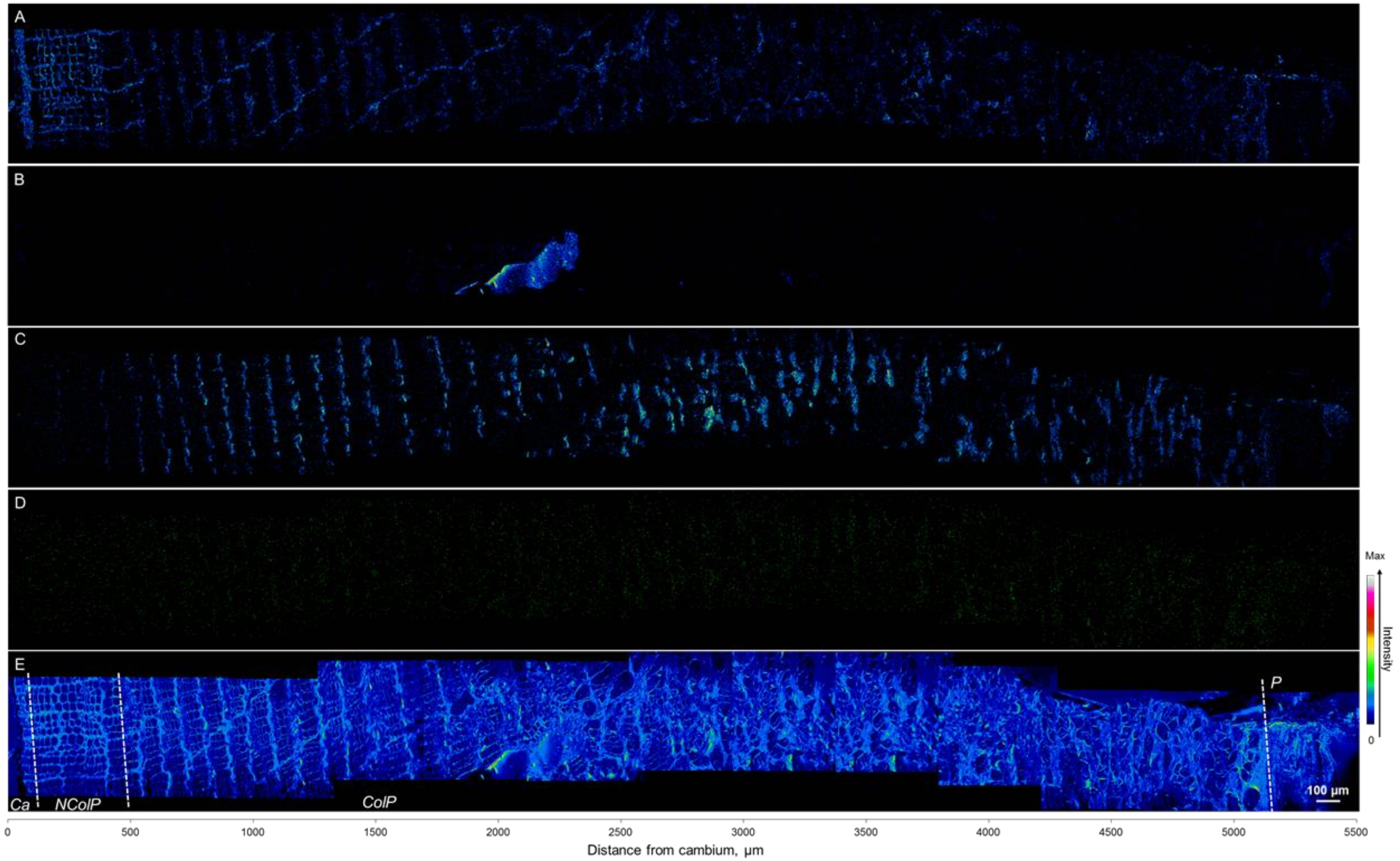

Figure S2. Positive ToF-SIMS images of transverse surface of Norway spruce phloem from the cambium (on left) to the outermost bark (on right) in a mature, non-wounded tree. Images of *m*/*z* at 184, 302, 245, and 291 representing living cells (A), abietic acid (B), stilbene glucoside astringin (C), and (+)-catechin (D), respectively. Total secondary ions (E) represent the structures of sample surface. The color of the pixels corresponds to the ion intensity of compounds on the specimen surface. Specifically, (+)-catechin and stilbenes were absent in dark areas, showed mid-range concentrations in blue areas, and had the highest concentration in green areas. Ca, cambium; NColP, non-collapsed phloem; ColP, collapsed phloem; P, periderm.

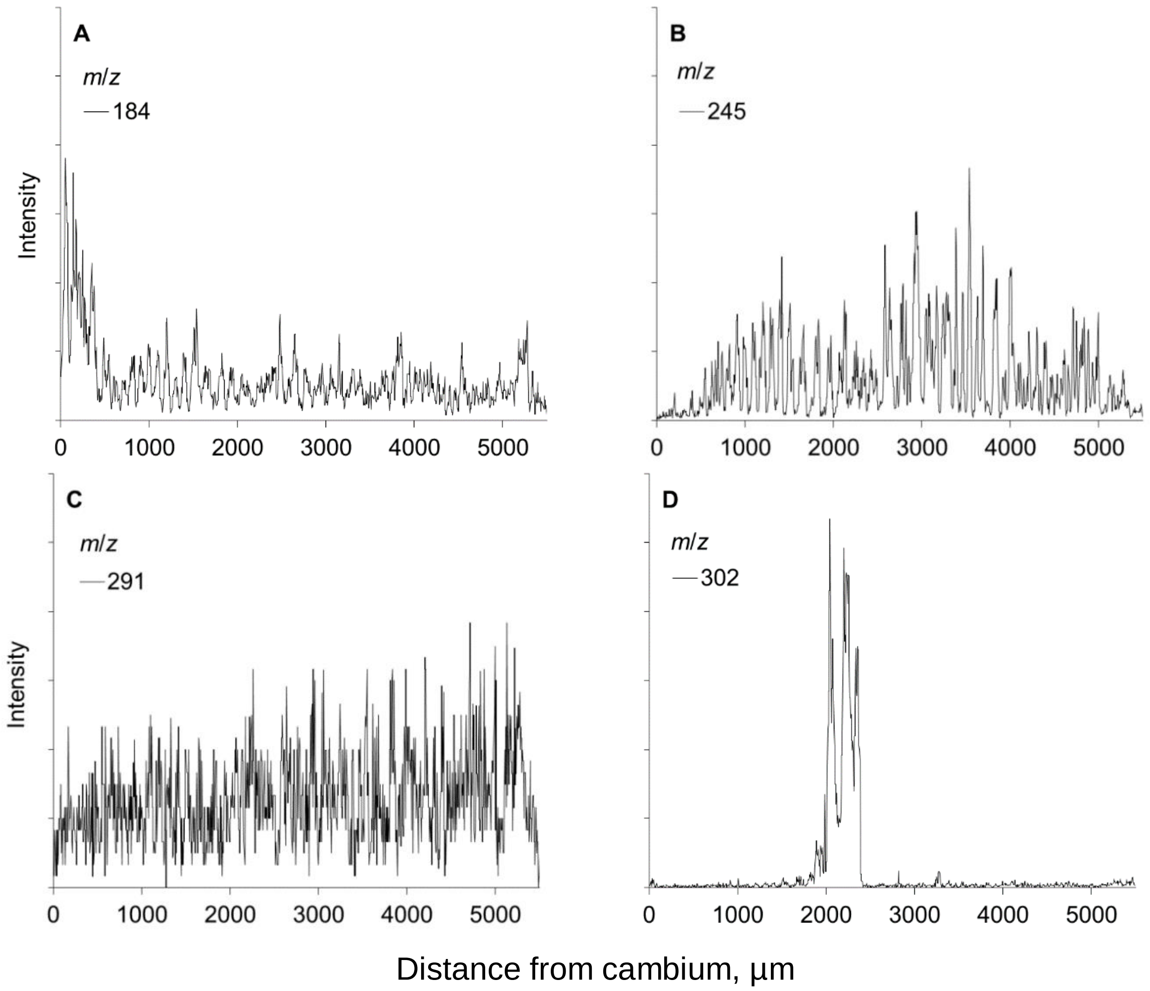

Figure S3. Ion intensities of *m*/*z* at 184, 245, 291, and 302 representing living cell membranes (A), stilbene glucoside astringin (B), (+)-catechin (C), and abietic acid (D), respectively, as obtained by ToF-SIMS spectra from the transverse surface of frozen-hydrated Norway spruce phloem of an older tree. Specimen is the same as that shown in Figure S2. Ion intensity values of three consecutive pixels were averaged and results shown as a function of distance from cambium.

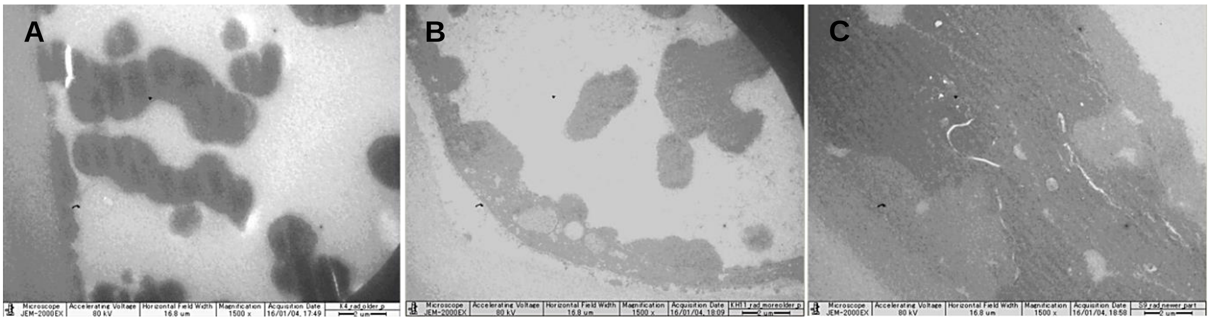

Figure S4. The TEM images representing typical electron dense (phenolic) contents of axial phloem parenchyma cells (obtained from tangential sections) of Norway spruce saplings: control (A), wounded (B), and fungal inoculated (C) specimen on the day 23 after treatment onset.

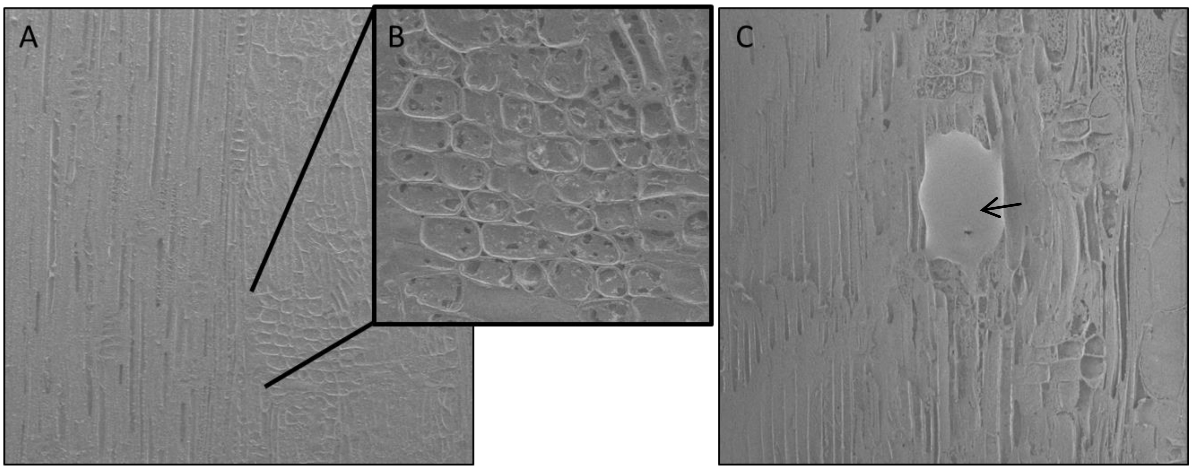

Figure S5. The cryo-SEM image obtained after cryo-ToF-SIMS analysis from a wounded (A, B) and fungal inoculated (C) sapling on day 23 after the treatment onset. B shows the structural details of parenchyma after freeze etching of the specimen. Outer bark is right from the imaged area. Arrow in C shows oleoresin droplet.
